# Micromorphology of Pine Needle Primordia and Young Needles after Bud Dormancy Breaking

**DOI:** 10.3390/plants12040913

**Published:** 2023-02-17

**Authors:** Marzenna Guzicka, Sławomir Marek, Magdalena Gawlak, Dominik Tomaszewski

**Affiliations:** 1 Institute of Dendrology of the Polish Academy of Sciences, Parkowa 5, PL-62-035 Kórnik, Poland; 2 Institute of Plant Protection—National Research Institute, Władysława Węgorka 20, PL-60-318 Poznań, Poland

**Keywords:** florin rings, micromorphology, phenological phases, *Pinus*, SEM, stomata

## Abstract

Using a scanning electron microscope, the micromorphologies of needle primordia and the young needles of seven pine species (*Pinus cembra*, *P. mugo*, *P. nigra*, *P. rigida*, *P. sylvestris*, *P. strobus*, and *P. uncinata*) were analyzed at phenological stages B2 and B3 (according to Debazac). In B2, needle tips were rounded or pointed, depending on the species. In *P. cembra* and *P. strobus*, teeth were noted on the tips. Teeth were also visible on the margins in *P. mugo*, *P. cembra*, and *P. strobus*. Stomata became visible in the late B2 phase (*P. sylvestris*, *P. mugo*, and *P. nigra*) near the needle tips and were arranged in rows. In the B3 phase, needle tips were pointed. Only in *P. strobus* was the needle tip slightly rounded. The teeth on the margin in all the species were pointed. In *P. strobus*, their size and density along the margin decreased basipetally. In B3 for all the species, numerous stomata were visible. In *P. sylvestris*, *P. cembra*, and *P. strobus*, Florin rings were also observed. These observations could be useful in pine systematics but also in palaeobotanical or physiological studies. To the best of our knowledge, this is the first study on the micromorphology of very young needles in representatives of the genus *Pinus*.

## 1. Introduction

Pines have a complex, polymorphic branch system consisting of long and short shoots, of which only the latter bear acicular leaves, called needles. The components of new shoots are defined during late summer and autumn preceding their appearance in the following spring as a series of primordia inside a bud in which they overwinter.

Long-shoot terminal buds in pines are divided into four zones: the zone of basal sterile scales, the zone of short-shoot buds with needle primordia and pollen cone buds, the zone of lateral buds or seed cone buds, and the zone of terminal scales with the shoot apical meristem [1,2,3,4,5,6]. The short-shoot zone is typically the largest and can cover up to 90% of the dormant bud length [4]. In the preceding season, the bud apical meristem forms a series of scales on the long shoot. The upper part of these scales withers and becomes brown, while the lower part remains green and alive [5].

Short shoots are covered by live cataphyll scales (typically 8–11), and the needle primordia of these shoots are tightly enclosed by them. Short-shoot buds typically contain all the needle primordia formed in autumn before bud dormancy [1,7]. In a long-shoot bud, there are primordia of each structure of the future mature shoot [7]. The growth of a shoot, defined as “predetermined” (buds contain all the primordia for the following season and, thus, the numbers of leaves and future lateral buds are already predetermined in the bud), can be completed in a relatively short time after dormancy breaking in spring [8,9]. The length of a shoot is, to a large extent, determined by the number of short-shoot primordia initiated in the previous season, which are present in the bud [10,11]. During the expansion of long-shoot buds into mature shoots, some phenological stages can be distinguished. Debazac [12] divided the process of growth into six phases, from B0 to B5, and proposed a classification consistent with bud morphology. These phenological stages were defined by him as follows: B0—dormant buds; B1—buds are swollen; B2—buds elongate and disjunction of bud scales makes the shoot surface visible; B3—emergence of brachyblasts, which remain entirely enveloped by the parts constituting the sheath; B4—emergence of needles; and B5—disjunction of needles from the same fascicle. 

Despite numerous works on the development of pine buds [5,6,13,14,15,16,17], only little attention is given to the morphology of needle primordia. Szubert [18], one of the precursors of research on Scots pines who studied bud and needle morphology, observed that, at first, growing needles are covered with delicate, semi-transparent scales, and white dots arranged in rows are visible on their surfaces. Initially, they were assumed to be resin glandules, but based on the illustration in Schubert’s work, these must be interpreted as stomata. General knowledge says that stomata are responsible for plant transpiration and gas exchange, playing an important role in water use efficiency and productivity [19]. However, despite the importance of stomata and numerous needle studies on pines, an important component of many types of forests in the world, little is known about the differentiation of the first stomata on pine needle primordia. 

In dormant winter buds, stomata are not visible on needle primordia [2], but they are on young needles after they emerge from buds. Most likely, as quickly as possible, the new needles start photosynthesis and, thus, effective gas exchange via the stomata is crucial. In pine seedlings, photosynthetically active cotyledons have stomata on both the adaxial and abaxial sides [20,21]. For growing needles, stomata in the B3 phase were shown in micrographs in the work of Dvořák and Štokrová [22]; however, in the descriptions, these structures were omitted by the authors. 

As photosynthetic organs, needles play a crucial role in ecophysiological research [23,24] and palaeobotanical studies [25]. The morphological traits of needles in pines, analyzed also as sets of traits, are used in the systematics of the group [26,27]. Stomata characteristics are important as a part of needle morphology, but despite this, little is known about them regarding needles still inside the bud. In this aspect, we think these characteristics may be important for researchers from various botanical disciplines.

Here, we are interested in answering a series of questions. It is known that needle primordia begin to lengthen before bud burst, but are the stomata on the needle surface already visible at that time? If they are, are they visible before the needle primordium starts growing in a dormant bud? How similar morphologically is a young needle inside a bud to a full-grown needle? Can the micromorphology of young needles be useful for systematics?

The aim of this work is to describe the micromorphologies of needle primordia and young needles inside buds for seven pine species in the B2 and B3 phenological phases according to Debezac [12]. Five of them belong to the subgenus *Pinus* (syn. *Diploxylon* (Koehne) Pilger), i.e., *Pinus mugo* Turra, *P. nigra* Arn., *P. rigida* Mill., *P. sylvestris* L., and *P. uncinata* (Ramond) Domin, and the other two, i.e., *P. cembra* L. and *P. strobus* L., belong to the subgenus *Strobus* (D.Don) Lemmon (syn. *Haploxylon* (Koehne) Rehder). To the best of our knowledge, this is the first report about the micromorphologies of needle primordium and young needles in these pine species.

## 2. Results

Needles of pines from the subgenus *Pinus* have semicircular or triangular cross-sections and are amphistomatic. *Pinus sylvestris*, *P. mugo*, *P. nigra*, and *P. uncinata* have two needles in a bundle, whereas *P. rigida* has three needles. The two species belonging to the subgenus *Strobus*, i.e., *P. cembra* and *P. strobus*, have five epistomatic needles per bundle, and their cross-sections are more or less triangular.

### 2.1. B2 Phase

**Figure 1:**
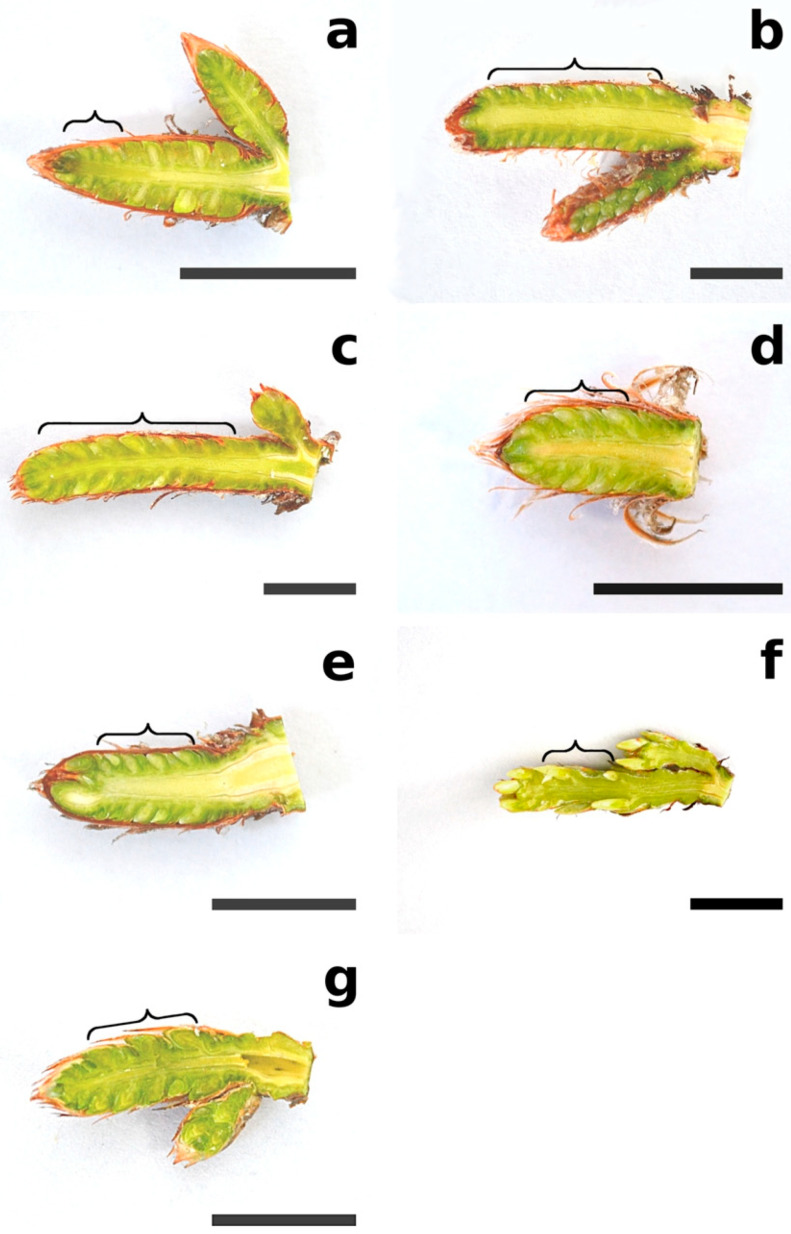
Longitudinal sections of long-shoot buds in B2 phase of growth: (**a**) *Pinus sylvestris*, (**b**) *P. mugo*, (**c**) *P. rigida*, (**d**) *P. nigra*, (**e**) *P. uncinata*, (**f**) *P. cembra*, and (**g**) *P. strobus*. Braces mark the short-shoot bud zones. Bar = 15 mm.

**Figure 2:**
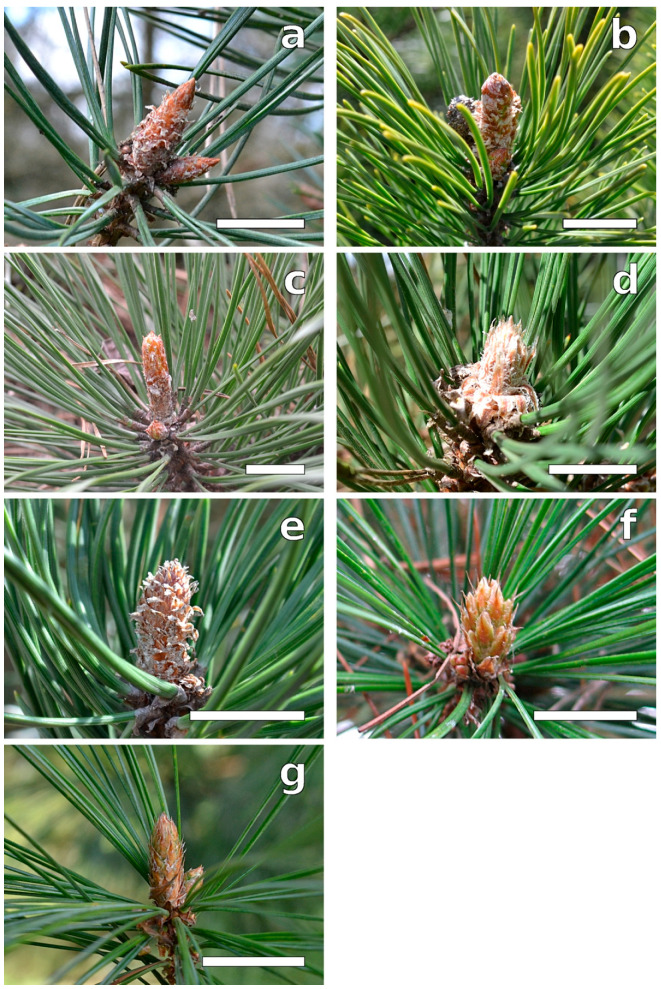
Buds during B2 phase: (**a**) *P. sylvestris*, (**b**) *P. mugo*, (**c**) *P. rigida*, (**d**) *P. nigra*, (**e**) *P. uncinata*, (**f**) *P. cembra*, and (**g**) *P. strobus*. Below buds, needles from the last year are visible. Bar = 15 mm.

**Figure 3:**
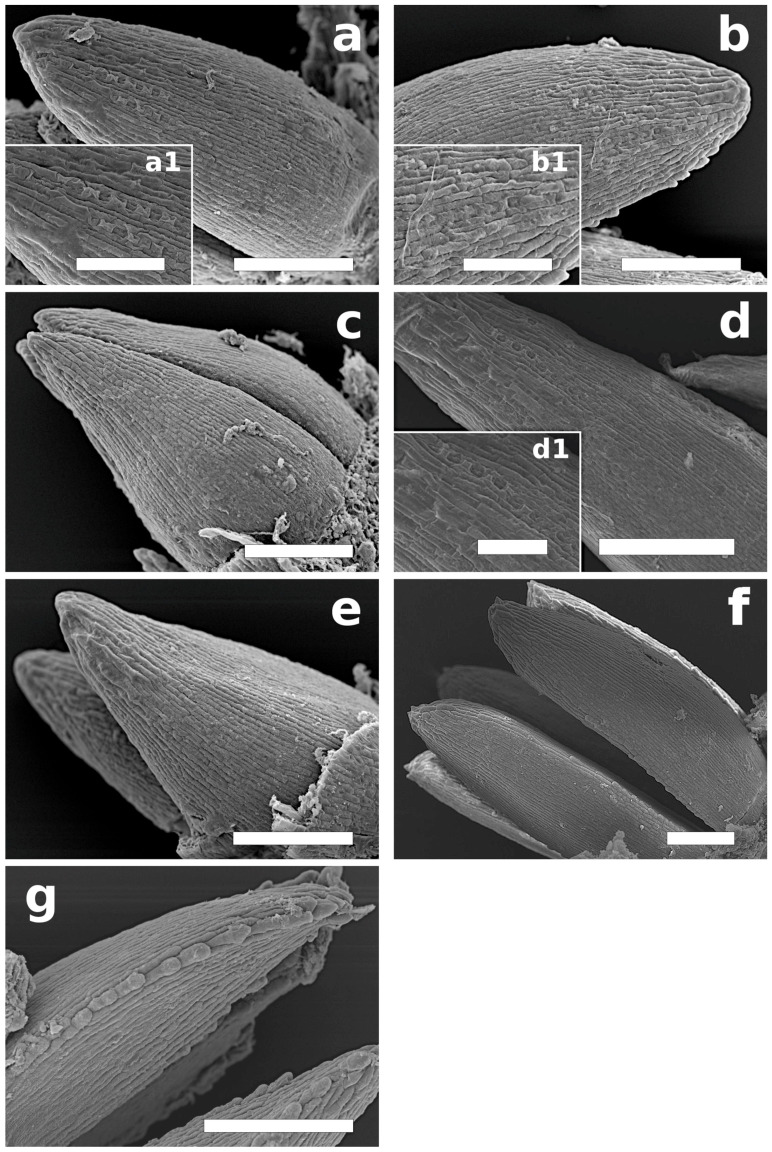
Needle primordia during B2 phase: (**a**) *P. sylvestris* needle primordium with smooth edges, slightly rounded apex, and stomata visible below the tip part of primordium; (**a1**) magnified part of (**a**); (**b**) *P. mugo* apex of needle primordium is rounded; small, pointed, irregularly dispersed teeth are seen on primordium edge, with the exclusion of top part; in close proximity to primordium apex, a few stomata can be observed; (**b1**) magnified part of (**b**); (**c**) *P. rigida* needle primordia with smooth edges, clearly pointed apexes, and no visible stomata on primordia surfaces; (**d**) *P. nigra* needle primordia with smooth edges and stomata visible in subapical region; (**d1**) magnified part of (**d**); (**e**) *P. uncinata* needle primordia with smooth edges and pointed apex, with no stomata visible; (**f**) *P. cembra* needle primordia with rounded apex and small, rounded teeth on needle edges, with no stomata visible; (**g**) *P. strobus* needle primordia with slightly pointed apex, densely distributed, rounded teeth on margin, and no stomata visible. Bar: (**a**–**c**,**e**–**g**) = 200 µm, (**d**) = 300 µm, and (**b1**,**d1**) = 100 µm.

During this phase, buds enlarged with disjunction of the scales, which then let tips appear between them and the surface of the twig (Figure 1 and Figure 2). Needle primordia were green (Figure 1). Two tip types were observed: rounded (*P. sylvestris*, *P. mugo*, and *P. cembra*) or pointed (*P. rigida, P. uncinata*, and *P. strobus*) (Table 1, Figure 3). In this phase, small teeth on needle primordia edges were present in three species: *P. cembra*, *P. mugo*, and *P. strobus* (Table 1, Figure 3). In *Pinus strobus*, teeth on the needle margin were numerous, wider in their top parts, and rounded; however, teeth were visible also on the needle tips, and these were pointed (Figure 3g).

The presence of stomata was noted in the upper part of needle primordia in three out of five representatives of the subgenus *Pinus* (*P. sylvestris*, *P. mugo*, and *P. nigra*) (Table 1, Figure 3a,b,d) but not in those of *Strobus*.

### 2.2. B3 Phase

**Figure 4:**
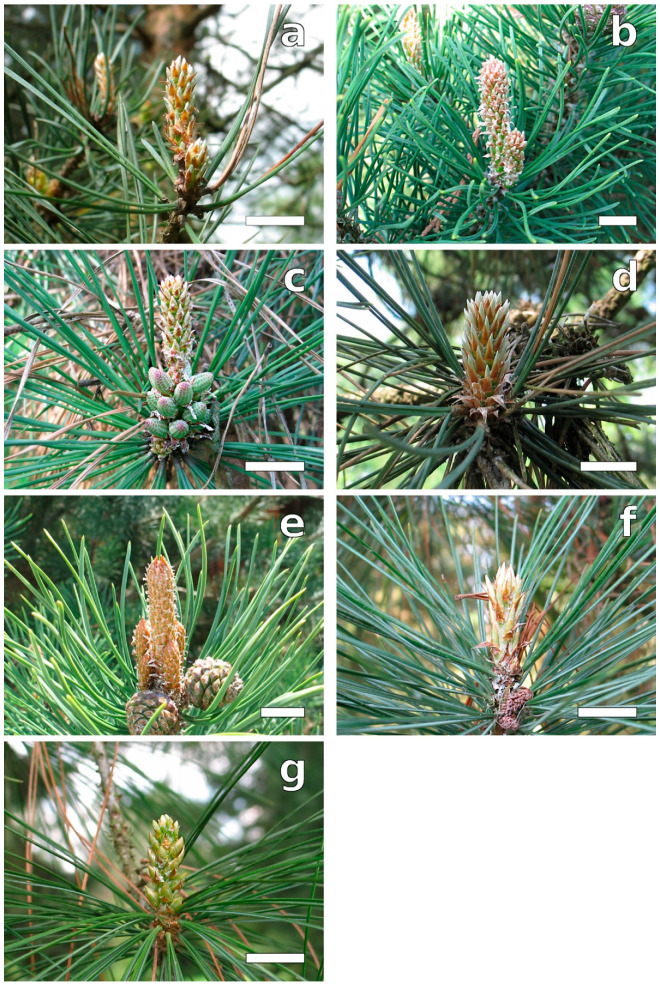
Buds during B3 phase. Young needles are tightly enclosed in cataphylls; below short-shoot zones, needles from the last year are visible. Short-shoot zone with pollen cones (**c**) or last year seed cones (**e**). (**a**) *P. sylvestris*, (**b**) *P. mugo*, (**c**) *P. rigida*, (**d**) *P. nigra*, (**e**) *P. uncinata*, (**f**) *P. cembra*, and (**g**) *P. strobus.* Bar = 15 mm.

**Figure 5:**
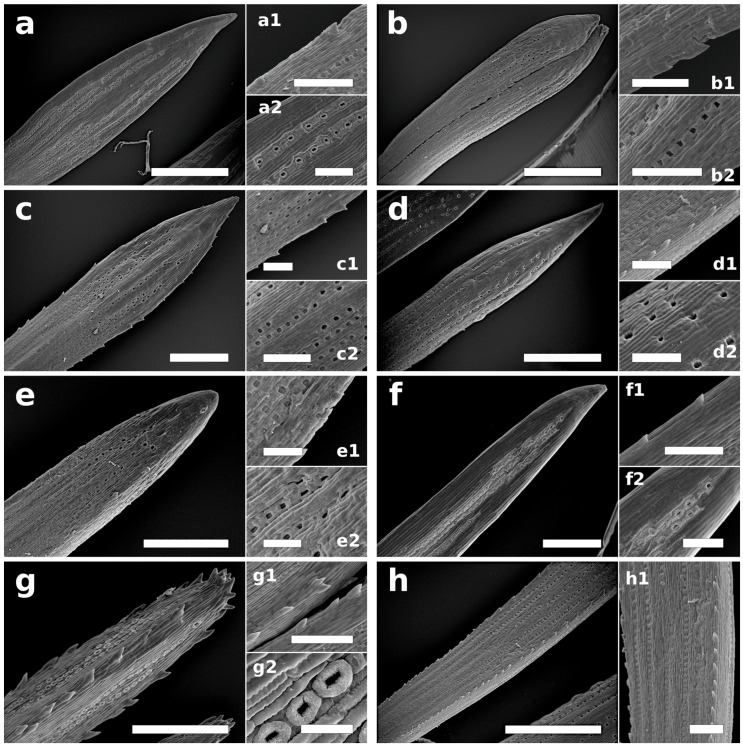
Young needles in B3 phase (SEM): (**a**–**a2**) *P. sylvestris*: (**a**) needle with pointed apex; rows of stomata are visible; (**a1**) on the edge of the needle, a small, pointed tooth is visible and fragments of two stomatal rows are visible, (**a2**) as well as two rows of stomata. (**b**–**b2**) *P. mugo*: (**b**) evenly dispersed teeth are visible on needle edge but not on needle tip; rows of stomata are well-developed; (**b1**) needle edge with pointed teeth and (**b2**) two rows of stomata. (**c**–**c2**) *P. rigida*: (**c**) needle with pointed apex; on needle edge, irregularly dispersed teeth and rows of stomata are clearly visible; (**c1**) needle edge with very small, pointed teeth and (**c2**) fragments of stomatal rows. (**d**–**d2**) *P. nigra*: (**d**) needle with long, pointed apex and stomatal rows are visible; (**d1**) needle edge with small, pointed teeth and (**d2**) fragments of stomatal rows. (**e**–**e2**) *P. uncinata*: (**e**) on abaxial side, needle apex is more or less pointed and rows of stomata are visible; (**e1**) needle edge with a few small teeth and (**e2**) rows of stomata. (**f**–**f2**) *P. cembra*: (**f**) needle apex slightly pointed with visible rows of stomata; (**f1**) needle edge with very small, pointed teeth and (**f2**) rows of stomata. (**g**–**g2**) *P. strobus*: (**g**) large, pointed teeth, located on needle edge as well as on rounded needle apex, and rows of stomata are visible; (**g1**) needle edges with large, pointed teeth and (**g2**) stomata with well-developed Florin rings. (**h**) *P. nigra* needle showing differences in development of stomata along needle axis, with basipetal direction of stomata development clearly visible; (**h1**) enlarged part of *P. nigra* needle showing stomata in early phase of differentiation. Bar: (**a**,**d**,**h**) = 1 mm; (**b**,**c**,**e**–**g**) = 500 µm; (**a1**–**d2**,**f1**–**g1**,**h1**) = 200 µm; (**e1**,**e2**) = 100 µm; (**g2**) = 50 µm.

**Figure 6:**
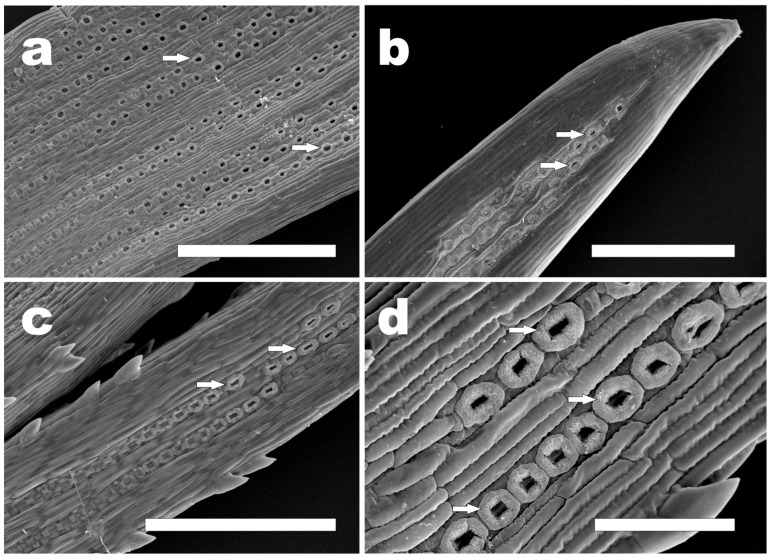
Young needles in B3 phase with Florin rings around stomatal pores (SEM): (**a**) *P. sylvestris* rows of stomata showing differences in development along needle axis; (**b**) *P. cembra*; (**c**) *P. strobus* rows of stomata showing differences in development along needle axis; (**d**) *P. strobus* stomata with well-developed Florin rings. Arrows point at some stomata with Florin rings. Bar: (**a**–**c**) = 500 µm; (**d**) = 100 µm.

In this phase, green needles remained entirely enclosed in cataphylls, although long-shoot bud scales had already lost their continuity, and short-shoot buds could be observed (Figure 4). The needles were usually narrower in the upper parts, except for *P. mugo* in which this part of the needle was wider, giving it a slightly club-like shape. The needles had a pointed tip shape typical for mature needles and pointed teeth on their edges (Table 1, Figure 5), except for *P. strobus* (Figure 5g). The latter species had teeth also on its slightly rounded needle tip—a trait not observed in other pines. In *P. mugo* and *P. nigra*, teeth on the needle margin were numerous. Especially numerous, big, pointed margin teeth were characteristic for *P. strobus* (Figure 5g,g1 and Figure 6c). Their size and density along the margin decreased basipetally.

In the B3 phase, numerous well-developed stomata were visible on needle surfaces in all the pines (Table 1, Figure 5). They were arranged in rows parallel to the needle longitudinal axis (Figure 5). It is worth mentioning that a stomata age gradient was observed (Figure 5h and Figure 6a,c). Stomata developed basipetally, i.e., from the needle tip to its base. In this phase based on stomata view, three distinct zones could be distinguished. In the upper parts of needles, well-developed stomata were found. In the middle parts, stomata in different developmental stages were visible (Figure 5f,g and Figure 6c), while in the lowest parts of the needles, stomata were not observed using SEM. In *P. sylvestris*, *P. cembra*, and *P. strobus* needles, in their upper parts well-developed stomata with clearly visible Florin rings were found (Figure 6).

**Table 1:** Morphological traits in chosen *Pinus* species in the B2 and B3 phases of bud development.

		Diploxylon					Haploxylon	
Needle Trait	Phase	*P. sylvestris*	*P. mugo*	*P. rigida*	*P. nigra*	*P. uncinata*	*P. cembra*	*P. strobus*
needle tip	B2	rounded	rounded	pointed	pointed	pointed	rounded, with very few teeth	pointed, with teeth
	B3	pointed	pointed	pointed	long and pointed	more or less pointed	pointed	slightly rounded, with teeth
teeth on needle margin	B2	not visible	small, pointed, near needle tip	not visible	not visible	not visible	few, rounded on the tip, pointed on base	numerous, rounded in upper part, pointed on tip
	B3	small, pointed, near needle apex	numerous, pointed	small and pointed, some near needle apex	numerous, small, pointed	few, small, pointed	few, small, pointed	numerous, big, pointed teeth covering needle tip
stomata	B2	few stomata near needle tip	few stomata near needle tip	not visible	few stomata near needle tip	not visible	not visible	not visible
	B3	numerous, arranged in rows, clearly visible Florin rings	numerous, arranged in rows	numerous, arranged in rows	numerous, arranged in rows	numerous, arranged in rows	numerous, arranged in rows, visible Florin rings	numerous, arranged in rows, visible Florin rings

## 3. Discussion

In all the pines species studied, we observed teeth on the leaf margins in the B3 phase (in *P. mugo*, *P. cembra*, and *P. strobus*, also in B2). In the five representatives of the subgenus *Pinus*, teeth on the leaf margins were always pointed, such as in mature needles, whereas in the two *Strobus* pines, in the B2 phase, teeth were rounded (in *P. strobus*, also wider in their upper part). In addition, only in *P. strobus* in B3 were numerous large, pointed teeth present on the needle tip. In this species, mature needles had finely serrulate margins, and the teeth were pointed. In the case of *P. strobus*, traits specific to mature needles regarding the tip and teeth took shape in phases later than B3. This is a unique feature of this pine. In the other six species, both the tips and margins of young needles inside the buds and mature needles were similar, and their final shapes were formed in the early stages of needle growth.

In needles, the morphology of stomata is of special interest. Stomata differentiate from protodermal cells. Protoderm, which differentiates into the epidermis, covers the surface of needle primordium [28]. In general, a protoderm lacks stomata. Some of its cells, stomatal progenitor cells (meristemoid mother cells), produce a stomatal complex. Stomata emerge from the protodermal cells as a protoderm differentiates into the epidermis. In gymnosperms, stomatal complexes are arranged in rows, and the rows present an age gradient, which is a consequence of divisions of the intercalary meristem located in the basal part of a needle [28]. The stomata are anatomical gates that allow plants to take up CO_2_, which is necessary for photosynthesis, while they retain a plant’s ability to control water loss by transpiration. Most of the water absorbed from the soil is released through the stomata, and therefore, stomata closing and opening are strictly connected to a plant’s response to environmental factors. The differentiation and development of stomatal complexes are closely tied to the need for gas exchange in growing needles [29]. In dormant winter buds, stomata are not visible on needle primordia [2], but they can be observed on young needles after they emerge from buds [4]. Stomata on growing pine needles in the B3 phase were described by Dvořák and Štokrová [22]. 

In our study, in the B2 phase (when bud enlarges with a disjunction of the scales, which then let the surface of the shoot appear between them), few stomata located on the apical parts of needles were noted in three species (*P. sylvestris*, *P. mugo*, and *P. nigra*), but their arrangement in rows was already visible. In the B3 phase, these were well-developed and arranged in rows (universal pattern of stomata distribution on needle surfaces in pines). In different parts of the needle, stomata at different developmental stages were observed. Although their initiation and development occurred in different needle parts, these processes happened simultaneously. Similar to *Tsuga heterophylla* [29] and *Pseudotsuga menziesii* [30], stomata were visible only in the uppermost part of the needles, and as the needles grew, the development of stomata proceeded basipetally, i.e., from the needle top to the base. 

The stomata present in phase B3 (when brachyblasts remained entirely enveloped by the parts constituting the sheath) in *P. sylvestris*, *P. mugo*, and *P. cembra* were arranged in the same pattern as those in mature needles. In physiological and eco-physiological studies, this distributional pattern is often used and can be valuable also in the case of very young needles, especially when we consider that the morphological and anatomical development of needles and leaves is correlated with the physiological status of a plant [23,31,32,33,34,35]. The presence of stomata in phase B2 and the development of stomatal complexes in phase B3 indicate that the differentiation of stomata began after the initiation of longitudinal needle growth but before the needles emerged from the bud in the late B2 phase. We should note here that stomata in the basal parts mature once needles completely emerge from buds, which is typical for conifers [8,36], but the number of stomatal rows is set in the early stages of needle growth inside buds [29]. Pine needles extend mainly by intercalary meristems located in the basal part [8], and this type of growth is characteristic for all gymnosperms [37].

Although some micromorphological traits were well-formed during the B3 phase, there was still an ongoing process of needle growth, and such traits as, for example, stomatal density (the number of stomata in a row per needle area) or the density of teeth on needle edges were not yet definitively established and could change with the elongation of the needle. In phase B3, in terms of many traits, young needles became similar to mature needles. Teeth were present on the needle edges in all the examined pines, and the needle apexes in this phase also corresponded with mature ones. The morphological characteristics typical of these species were visible at an early stage. It must be noted that needle margin (smooth or serrated) is a trait often provided in taxonomic keys [38]. Needle morphology, including needle margins with or without teeth, is also considered in the study of fossil material, and such studies could also have a biogeographic implication [39]. Cuticular features are used to help to determine the relationships of taxonomically difficult taxa [40]. Specific needle epidermal features, including stomata apparatus characteristics, contain some important information for identifying pines of native southwestern European [41], Mexican, Central American [40], and North American [42] origins. Analyses of contemporary species could be useful to identify isolated cuticles and stomata in palynological slides. 

In some cases (*P. cembra*, *P. strobus*, and *P. sylvestris*), we observed Florin rings on the needles, which are thick, cuticular ridges commonly formed around the stomatal pit as a coalescent structure over the proximal walls of subsidiary cells [24]. In other words, Florin rings are “conspicuous thickened rings of cutin overlying the accessory cells and surrounding the stomata” [43]. They are associated with the subsidiary cells observed in both living and fossil gymnosperms, such as *P. strobus* or *Papuacedrus* [44,45], and were also described in *Falcatifolium* (Podocarpaceae) [46].

These structures were described for the first time by Florin [47], who also drew attention to their importance for taxonomy. The value of this trait has also been emphasized by other authors [48,49]. Yoshie and Sara [50] studied the shapes of Florin rings in 51 species in the genus *Pinus* and classified them. Six different types of Florin rings have been described, four of which are seen in the subgenus *Pinus* [50,51]. In some cases, species can be identified by Florin rings, which was suggested in taxonomic studies based on needle epidermis features [41]. Florin rings are particularly useful for infrageneric classification [40,42,52].

In the cases of many fossilized conifers, often only little parts of vegetative organs (e.g., small pieces of needles) are available. Analyses of the cuticles in living and fossil gymnosperms have shown that stomatal and other epidermal characteristics are often of great value in the delimitation of genera, as well as in distinguishing allied species by fragmentary fossil remains (see [47,53,54]). At times, isolated stomata may appear in palynological preparations [55]. In this context, stomata analysis is important not only in modern taxonomic studies [56] but also as a tool for evolutionary, palaeoecological, and palaeoenvironmental research [57,58,59,60,61] and, together with plant macrofossils, may thus refine pollen-inferred reconstructions. Our observations of well-developed Florin rings showed that even very young needles (B3 phase) have the potential to be useful in this kind of research. 

## 4. Materials and Methods

The morphologies of needle primordia and young needles of seven pine species were examined. All plant material (Table 1) was collected from the Arboretum of the Institute of Dendrology in Kórnik, Poland (52°14′40.2″ N, 17°05′27.5″ E). The first sampling of apical buds was conducted from April 7 to 18 during phase B2 of bud development [12]. The second sampling was conducted from April 25 to 29 during the B3 phase. Very young needles were isolated from short-shoot buds located in the basal parts of long-shoot primordia in buds (Figure 1). 

Isolation was conducted in a drop of 0.05 M cacodylic buffer to prevent samples from drying. Needles and needle primordia were observed and imaged with a stereomicroscope (Nikon SMZ 800, Tokyo, Japan) and a scanning electron microscope (SEM) (Hitachi S3000N, Institute of Plant Protection—National Research Institute) equipped with a secondary electron detector. Material for SEM studies was fixed in 4% glutaraldehyde and 4% paraformaldehyde in 0.1 M cacodylic buffer (pH 6.9), post-fixed using 1% OsO_4_ and dehydrated in an increasing ethanol solution series [62]. The specimens were then critical-point dried, mounted on aluminium stubs using double-sided adhesive carbon disks, and coated with gold and palladium.

## 5. Conclusions

We analyzed needle primordia and young needles of seven pines species after bud dormancy breaking. In all of them, needle primordia started their growth inside the bud, and the growth of the leaf primordia led to bud bursting. At that time, their morphological traits, such as the shape of the needle tip, the margin, and the stomata, were different from those of mature needles. Differentiation of the stomata from protodermal cells started inside the bud after the initiation of needle growth in the late B2 phase. In B2, few stomata were visible only at the needle tips of *P. sylvestris*, *P. mugo*, and *P. nigra*, but in those early stages, their arrangement in rows was already settled. In phase B3, more traits of young needles were similar to those of mature needles. Teeth were present on the needle edges in all the species, and the shapes of the needle tips were similar to those in mature ones. Stomata were visible in all the species in the B3 phase and were arranged in the same pattern as in mature needles; in some cases, Florin rings were visible. Particularly noteworthy was the micromorphology of young needles of *Pinus strobus* because, unlike the other six species, in B3 young needles differed from mature needles.

To the best of our knowledge, our report provides the first information on the micromorphologies of needle primordia and young needles in representatives of the genus *Pinus*. In our opinion, the information that stomata were clearly visible in young needles in the late B2 or B3 phases could be important in physiological studies of bud development, analyses of photosynthesis and respiration in spring bud, or research on the carbon balance in buds. We also think that the presence of well-developed Florin rings in B3 may allow using very young needles in palaeobotanical studies as material for comparison with fossilized needles.

## Data Availability

The data presented in this study are available on request from the corresponding authors.
